# Literary Workers

**Published:** 1906-09-01

**Authors:** 


### LITERARY WORKERS.

Sometimes a medical man aspires to contribute to the literature of the profession, or at least to augment his income by devoting his leisure time to medical literary work. It is very hard, opportunities of success rare, and often fraught with disappointment. It has advantages, however, in keeping the willing worker in touch with the progressive students of medical science. The medical profession is exceedingly rich in having in its ranks many very good writers, both on professional and lay subjects. Medical men are obliged to keep their medical capital in a state of constant growth by means of recent book and magazine literature. There are many valuable libraries open to medical men. The library of the Royal College of Surgeons, Lincoln's Inn Fields, is open to all practitioners who have a card of introduction from a fellow or member. The library of the British Museum is available at all times. The library of the Royal Medical and Chirurgical Society may be available after an introduction by a Fellow; and the library of the Royal College of Physicians may be available on certain easy conditions. The valuable library of the Royal College of Physicians of Edinburgh may be used by suitable persons.

